# Knockdown of RSPH14 inhibits proliferation, migration, and invasion and promotes apoptosis of hepatocellular carcinoma via RelA

**DOI:** 10.1186/s12935-022-02515-z

**Published:** 2022-03-19

**Authors:** Dawei Yuan, Rulan Ma, Tuanhe Sun, Kun Zhu, Chengxue Dang, Haixia Ye, Kang Li

**Affiliations:** 1 Department of Surgical Oncology, The First Affiliated Hospital of Xi’an Jiaotong University, 277 West Yanta Road, Xi’an, 710061 Shaanxi People’s Republic of China; 2 School of Basic Medical Sciences, Wuhan University, Wuhan, 430071 Hubei People’s Republic of China

**Keywords:** Hepatocellular carcinoma, RSPH14, RelA, Proliferation, Migration

## Abstract

**Background:**

High RSPH14 expression appears to be related to poor prognosis of hepatocellular carcinoma (HCC). This study aimed to investigate the possible roles of RSPH14 in the proliferation, apoptosis, and invasion of HCC cells.

**Methods:**

The UALCAN database and Kaplan–Meier Plotter were used to evaluate the expression level and prognostic role of RSPH14 in HCC. Lentiviral vectors containing shRNA against RSPH14 were constructed to transfect the BEL-7404 and SMMC-7721 HCC cell lines. Cell proliferation was investigated by BrdU, MTT, and colony-formation assays. Apoptosis was detected using flow cytometry. Cell migration and invasion were evaluated using the scratch wound-healing and Transwell assays. Immunohistochemistry and western blot were used to determine the expression levels of the proteins. The function of RSPH14 in vivo was evaluated using a xenograft mouse model.

**Results:**

The expression of RSPH14 was higher in HCC tumor tissues than in adjacent normal tissues and was closely related to unfavorable prognostic factors and poorer survival (all P < 0.05). Knockdown of RSPH14 inhibited the cell proliferation, migration, and invasion of HCC cells and promoted apoptosis (all P < 0.05). Knockdown of RSPH14 inhibited tumor growth in vivo (P < 0.05). RSPH14 knockdown led to decreased expression of RelA (NF-κBp65), CDH2, and AKT1, thereby affecting the functions of the HCC cells (all P < 0.05). RelA overexpression could abate the inhibitory effect of BEL-7404 cell proliferation caused by RSPH14 depletion.

**Conclusion:**

Knockdown of RSPH14 could decrease cell proliferation, migration, and invasion and increase apoptosis of HCC cells by inhibiting RelA expression. RSPH14 could be a new treatment target for HCC.

**Supplementary Information:**

The online version contains supplementary material available at 10.1186/s12935-022-02515-z.

## Introduction

Primary liver cancer is the sixth most common malignant tumor and the third leading cause of cancer death worldwide in 2020, with 905,677 new diagnostic cases and 830,180 deaths [1]. In 2021, 42,230 new cases and 30,230 deaths from liver cancer are projected to occur in the United States [2]. Primary liver cancer includes hepatocellular carcinoma (HCC), intrahepatic cholangiocarcinoma, and other rare types; 75–85% of primary liver cancers are HCC. The main treatment strategies for early-stage HCC include surgery, radiotherapy, chemotherapy, and palliative therapies such as radiofrequency ablation [3]. On the other hand, therapeutic strategies for patients with advanced HCC are limited [4]. Therefore, it is urgently needed to improve our understanding of HCC pathogenesis and explore new feasible targets for treatment strategies to improve the poor prognosis of HCC patients.

Radial Spoke Head 14 Homolog (RSPH14), also called rhabdoid tumor deletion region gene 1 (RTDR1), is located in a region deleted in pediatric rhabdoid tumor of the brain, kidney, and soft tissues. RSPH14 is a novel gene that encodes a protein whose function is unknown but slightly similar to the yeast vacuolar protein [5]. RSPH14 is recognized as a prognostic marker in renal cancer [6]. Based on the Human Protein Atlas data, a high expression level of RSPH14 is associated with a poor prognosis of patients with liver cancer [6]. Still, the functions of RSPH14 in HCC progression and prognosis, as well as its mechanism, are unknown.

Studies published in the past few years demonstrate that several signaling pathways play important roles in the proliferation of HCC cells, including ERK/p38MAPK [7, 8], AKT [7, 8], and NF-κB [9, 10]. Myc is an apoptosis-promoting gene that contributes to liver cancer cell apoptosis [11, 12]. In addition, the epithelial to mesenchymal transition (EMT) is closely related to the migration and invasion of HCC [13, 14]. The molecular markers of EMT include E-cadherin (CDH1), N-cadherin (CDH2), β-Catenin, Slug, Snail, Twist, MMP2, Vimentin, and FN1 [15, 16].

Since the mechanisms responsible for RSPH14-associated poor prognosis of HCC are mostly unknown, this study aimed to investigate the possible roles of RSPH14 in the proliferation, apoptosis, and invasion of HCC cells. The results could provide additional insights into the pathogenesis of HCC and help identify possible treatment targets.

## Methods

### Gene expression analysis

The expression analysis of RSPH14 in HCC tumor tissue and adjacent normal tissue was performed using the UALCAN database (http://ualcan.path.uab.edu/) [17]. Kaplan–Meier Plotter (http://kmplot.com/analysis/) [18] was used to evaluate the overall survival rate (OS) and progression-free survival (PFS) of the patients with HCC according to the expression level of RSPH14.

### Cell culture

The HCC cell lines BEL-7404 and SMMC-7721 were purchased from the Cell Bank of the Chinese Academy of Sciences (Shanghai, China). All cells were cultured at 37 °C with 5% CO_2_ in Dulbecco’s modified Eagle’s medium (DMEM, Corning, 10-013-CVR) with 10% fetal bovine serum (FBS, VS500T, Ausbian, Shanghai, China).

### Transfection

The shRNA sequence (5′-GAT CAT CAG CAA AGG TCT GAT-3′) was designed for targeting RSPH14, whereas a scrambled sequence (5′-TTC TCC GAA CGT GTC ACG T-3′) was set as a negative control. The single-stranded DNA oligo chain containing the interference sequence was synthesized, followed by the double-stranded DNA. The restriction enzyme cleavage site was ligated to the lentiviral vector. Competent *E. coli* was transformed with the ligation vector. The positive transformants were identified and verified, and the lentiviral vectors with the correct sequences were extracted and purified.

The lentiviral vectors containing shRNA against RSPH14 were transfected into BEL-7404 and SMMC-7721 cells. The medium was changed at 16 h after transfection. The expression of the green fluorescent protein was detected by a fluorescence microscope (Olympus, IX71) at 72 h after infection. When the efficiency of cell infection exceeded 80%, and the degree of cell confluence reached 80%, the cells were collected for further study.

### BrdU assay for cell proliferation

The BEL-7404 and SMMC-7721 cells were infected with the lentiviral vector containing the shRNA against RSPH14 (KD group) or the negative control vectors (NC group) for 3 days. Then, the infected cells were seeded into 96-well plates (2000 cells/well) and incubated at 37 °C with 5% CO_2_ in DMEM (10-013-CVR, Corning Inc., Corning, NY, USA) with 10% FBS (VS500T, Ausbian). A BrdU kit (11647229001, Roche Applied Science, Penzberg, Germany) was used to evaluate cell proliferation, according to the manufacturer’s instructions. The absorbance at 450 nm was measured using a microplate reader (M2009PR, Tecan Group Ltd., Mannedorf, Switzerland).

### Celigo cell counting

The transfected cells were seeded into 96-well plates (1500 cells/well) and incubated at 37 °C with 5% CO_2_ in DMEM (10-013-CVR, Corning) with 10% FBS (A11-102, Ausbian) for 5 days. The Celigo image cytometry system (Nexcelom Bioscience LLC, Lawrence, MA, USA) was used to determine the cell count. From the second day after seeding, the cell count was measured each day for 5 consecutive days. Cell images with green fluorescence were analyzed by the Celigo software (Nexcelom).

### MTT assay

The transfected cells were seeded into 96-well plates (2000 cells/well). Then, 24 h later, the cells were added with 20 µl of 5 mg/ml MTT solution (JT343, Genview, Galveston, TX, USA) and incubated for 4 h. The medium was discarded, and 100 µl of dimethyl sulfoxide solution (Shanghai Shiyi Co., Ltd, 130701) was added. The plates were shaken for 2–5 min, and the absorbance at 450 nm was measured using a microplate reader (M2009PR, Tecan infinite).

### Colony formation assay

The BEL-7404 and SMMC-7721 cells were transfected for 3 days. The infected cells were seeded into 6-well plates (800 cells/well) and cultured for 13 days. The culture medium was changed every 3 days. The cells were washed with PBS once and fixed with 1 ml of 4% paraformaldehyde (SINOPHARM, Beijing, China) for 30–60 min. The cells were washed with PBS once and stained with 1000 µl of methyl violet (Sangon Biotech, CB0331) for 10–20 min. The cells were washed with ddH_2_O several times and dried at room temperature. The cell colonies were scanned by a microscope (Cai Kang Optical Instrument Co., Ltd, XDS-100).

### Cell apoptosis

The transfected cells were seeded into 6-well plates and incubated for 5 days. Cell apoptosis was assessed using an Annexin V-APC Apoptosis Detection kit (88–8007, eBioscience, San Diego, CA, USA), according to the manufacturer’s instructions. The cells were stained with Annexin V-APC, and the number of apoptotic cells was detected by flow cytometry (Guava easyCyte HT, Millipore Corp., Billerica, MA, USA).

### Scratch wound-healing assay

The transfected cells were seeded in 6-well plates, and the cell density was exceeded 90%. Then, 24 h later, scratches were drawn across the plate with a Wounding Replicator (VP408FH, V&P Scientific, Inc., San Diego, CA, USA), and the cells were washed with 2% PBS 2–3 times. Images were scanned at 0, 18, and 36 h using the Celigo image cytometry system (Nexcelom) after 1% FBS was added into the wells. The migratory rate was calculated using the equation: migratory rate (%) = (St − S0)/S0 × 100%, where S0 is the initial migration area, and St is the migration area at 18 and 36 h.

### Transwell migration and invasion assays

The migration assay was performed using the Transwell migration kit (3433, Corning), according to the manufacturer’s instructions. Briefly, 1 × 10^5^ BEL-7404 and SMMC-7721 cells in 100 µl were seeded into the apical chamber, and 600 µl of 30% FBS was added into the basolateral chamber of each well. The cells were incubated at 37 °C with 5% CO_2_ for 48 h. The medium was discarded, and the non-migrated cells were swabbed. The chambers were fixed with 4% paraformaldehyde (Sinopharm Chemical Reagent Co., Ltd.) for 30 min. The cells were stained with Giemsa dye (32884, Sigma, St. Louise, MO, USA) for 1–3 min. The cell count was measured under a microscope (IX71, Olympus) at 200× magnification, and an average of 9 fields per chamber was assessed.

The Transwell invasion kit (354480, Corning) with Matrigel matrix was used to evaluate the invasion ability of the cells. The apical chamber and the basolateral chamber were added with 500 µl of medium and incubated at 37 °C with 5% CO_2_ for 2 h. The medium of the apical chamber was discarded, and 200 µl cell suspension (1 × 10^5^ transfected cells) was added into the apical chamber. The basolateral chambers were added with 750 µl of 30% FBS. The cells were incubated for 48 h. The medium was discarded, and the non-migrated cells were swabbed. The cells were stained with Giemsa dye (32884, Sigma) for 2–3 min. The cell count was measured under a microscope (IX71, Olympus) at 200× magnification, and an average of 9 fields per chamber was assessed.

### Immunohistochemistry

The RSPH14 protein levels were detected in 28 HCC and the corresponding normal tissues using immunohistochemistry. Tissue specimens were embedded in paraffin, and serial 4-mm sections were cut, deparaffinized, and rehydrated. The sections were incubated with a primary rabbit anti-human RSPH14 antibody (PA5-113475, 1:200 dilution, Invitrogen Inc., Carlsbad, CA, USA) at 4 °C overnight. They were then incubated with the secondary antibody (goat anti-rabbit IgG, 1:200 dilution, Pierce antibodies, Thermo Fisher Scientific, Waltham, MA, USA) for 60 min at room temperature, and 3,3′-diaminobenzidine was used as the chromogen. The slides were counterstained with hematoxylin solution and dehydrated and slip-covered. Images of stained sections were obtained using an optical microscope (BX51, Olympus) equipped with a digital camera (PD71, Olympus). RSPH14 was scored by the percentage of positive tumor cells: 0, < 15%; 1+, 16–30%; 2+, 31–60%; and 3+, 61–100%. All stained sections were independently evaluated by two investigators, and agreement was reached after careful discussion if discrepancies occurred.

### Western blot

The proteins were extracted from the cells using a RIPA buffer. The concentrations of proteins were measured by a BCA Protein Assay Kit (P0010S, Beyotime Institute of Biotechnology, Haimen, China). Western blotting was performed to analyze the isolated proteins. The antibodies used in this assay included anti-rabbit-RSPH14 (A15430, dilution 1:200, Abclonal Technology, Woburn, MA, USA), anti-GAPDH (sc-2004, dilution 1:2000, Santa Cruz Biotechnology, Santa Cruz, CA, USA), anti-Vimentin (ab92546, dilution 1:500), anti-Slug (#9585, dilution 1:1000, Cell Signaling Technology, Inc., Danvers, MA, USA), anti-Snail (#3879, dilution 1:500, CST), anti-Myc (ab18203, dilution 1:500, Abcam), anti-Twist (ab50887, dilution 1:50, Abcam), anti-FN1 (AB6328, dilution 1:1000, Abcam), anti-RelA (#8242, dilution 1:500, CST), anti-p-RelA (#3033, dilution 1:1000, CST), anti-P38mapk (#8690, dilution 1:500, CST), anti-p-P38mapk (ab4822, dilution 1:500, Abcam), anti-β-Catenin (#8480, dilution 1:500, CST), anti-p-β-Catenin (#2009, dilution 1:1000, CST), anti-ERK1/2 (#9107, dilution 1:300, CST), anti-p-ERK1/2 (#4376, dilution 1:300, CST), anti-AKT1 (#4691, dilution 1:500, CST), anti-p-AKT (#4051, dilution 1:500, CST), anti-MMP2 (ab37150, dilution 1:1000, Abcam), anti-CDH1 (ab1415, dilution 1:50, Abcam), anti-CDH2 (ab92546, dilution 1:100, Abcam), anti-Rabbit-IgG (sc-2004, dilution 1:2000, Santa Cruz), and anti-Mouse IgG (sc-2005, dilution 1:2000, Santa-Cruz,) antibodies.

### In vivo experiment

Six- to eight-week-old female BALB/c-nu nude mice (n = 6/group) were obtained from the Animal Experimental Center of Xi’an Jiaotong University (Xi’an, China). No randomization was used when animals were selected. All animal experiments were approved by the Institutional Animal Care Committee and met the standards set by the Ethics Committee of Experiment Animals. The animals were fed in an air-conditioned room with abundant water and oxygen. The animals were randomly divided into two groups. The BEL-7404 cells with or without RSPH14 knockdown (1 × 10^6^) were injected subcutaneously in the mice’s right flanks. PBS alone were subcutaneously injected in their left flanks. The weight of the mice was recorded every 7 days. Tumor growth was assessed every 5 days using Vernier calipers for the following 30 days, and the volume was calculated as tumor volume (mm3) = (length × width2)/2. On day 30 after injection, the mice were put into the euthanasia box and euthanized by perfusing CO_2_ into the euthanasia box at a flow rate of 10–30% for 5 min.

### Statistical analysis

GraphPad Prism 8.3.0 (GraphPad Software Inc., San Diego, CA, USA) and SPSS 22.0 (IBM Corp., Armonk, NY, USA) were used for data analysis. Data were presented as mean ± standard error of the mean (SEM). Statistical differences were calculated by t-test or repeated-measures analysis of variance. Differences with P-values < 0.05 were considered statistically significant.

## Results

### RSPH14 is highly expressed in HCC and related to a poor prognosis

**Fig. 1:**
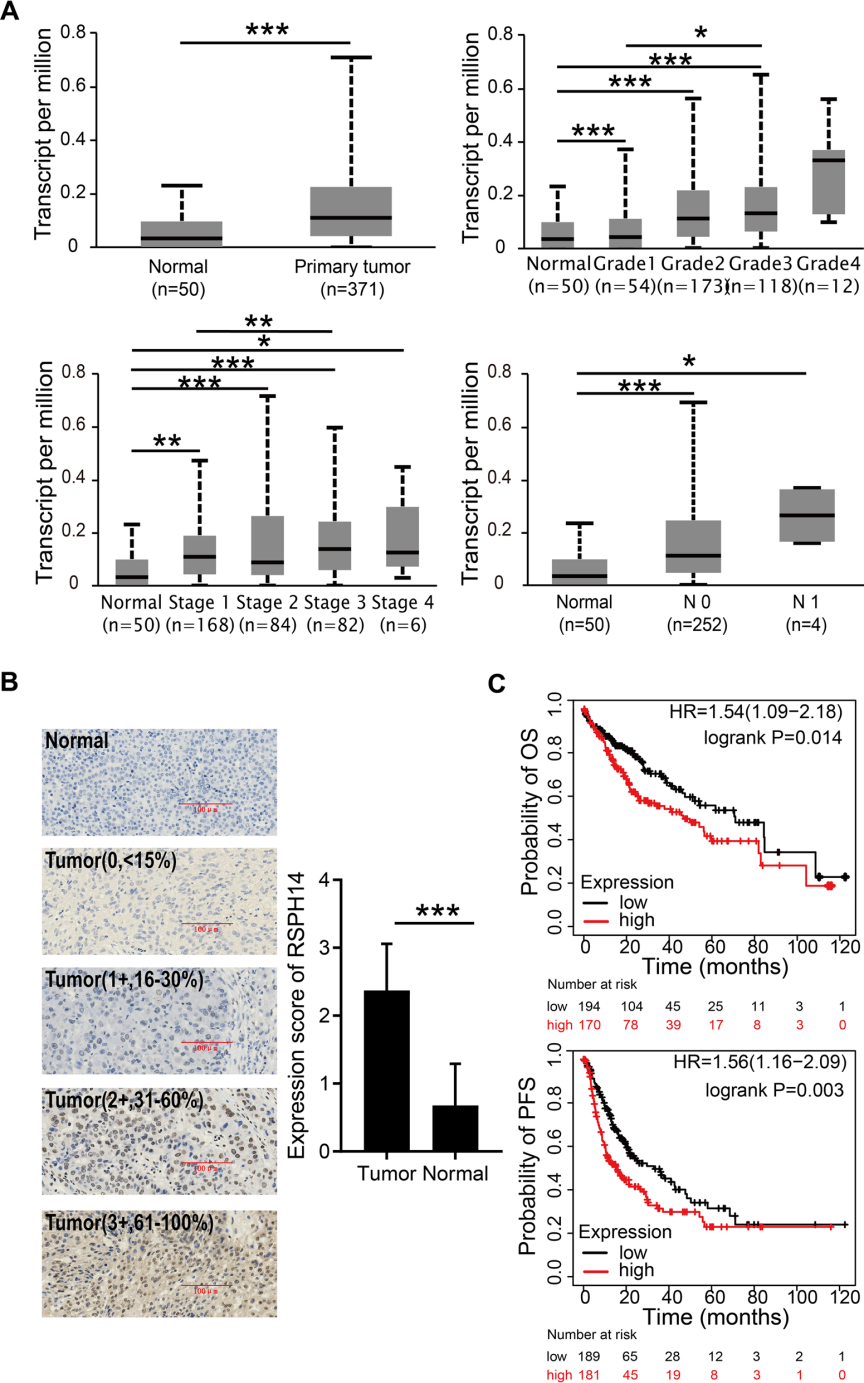
The expression level of RSPH14 and its relationship with survival rates in HCC. **A** The expression levels of RSPH14 in tumor tissues and adjacent normal tissues in HCC by UALCAN database. **B** Representative IHC images showing the different staining patterns of RSPH14 expression (scored by the percentage of positive tumor cells: 0, < 15%; 1+, 16–30%; 2+, 31–60%; and 3+, 61–100%). Scale bar, 100 µm. **C** Survival analysis of RSPH14 expression in HCC patients by Kaplan–Meier analysis. HCC: hepatocellular carcinoma; OS: overall survival; PFS: progression free survival. *P < 0.05, **P < 0.01, ***P < 0.001

In order to investigate the expression of RSPH14 in HCC tissues and adjacent normal tissues, we retrieved data from the UALCAN database and found that the expression level of RSPH14 was higher in tumor tissues compared with adjacent normal tissues in HCC patients (P < 0.05) (Fig. 1A). Notably, the expression level of RSPH14 increased with the increase of tumor differentiation degree, lymph node metastasis, and tumor stage (all P < 0.05) (Fig. 1A). In addition, we detected the RSPH14 protein level in a total of 28 HCC and the corresponding normal tissues using immunohistochemistry. The results showed that RSPH14 was elevated in human HCC tissues compared with the paired non-cancerous tissue (P < 0.001) (Fig. 1B). We further evaluated the prognostic role of RSPH14 in patients with HCC. The results of survival analysis performed using the Kaplan–Meier plotter database showed that the OS and PFS of the patients with lower RSPH14 expression were significantly higher, comparing to the patients with higher RSPH14 expression (HR = 1.54, P-value = 0.014; HR = 1.56, P = 0.003) (Fig. 1C). These results suggest that the expression of RSPH14 is higher in tumor tissues of HCC and is associated with the poor prognosis of HCC patients.

### RSPH14 knockdown in HCC cell lines using shRNA

Due to the high expression level of RSPH14 in HCC tissues and its relationship with poor clinical outcome, we constructed the lentivirus containing shRNA for targeting RSPH14 and transfected BEL-7404 and SMMC-7721 cells with the lentiviral vectors to downregulate RSPH14 expression to investigate the role of RSPH14 in HCC. The expression of green fluorescent protein showed the efficiency of cell transfection (Additional file 1: Fig. S1A). Moreover, western blotting showed that, after lentiviral infection, RSPH14 was significantly silenced in both BEL-7404 and SMMC-7221 cells (Additional file 1: Fig. S1B).

### RSPH14 knockdown restrains proliferation and promotes apoptosis of HCC cells in vitro and in vivo

**Fig. 2:**
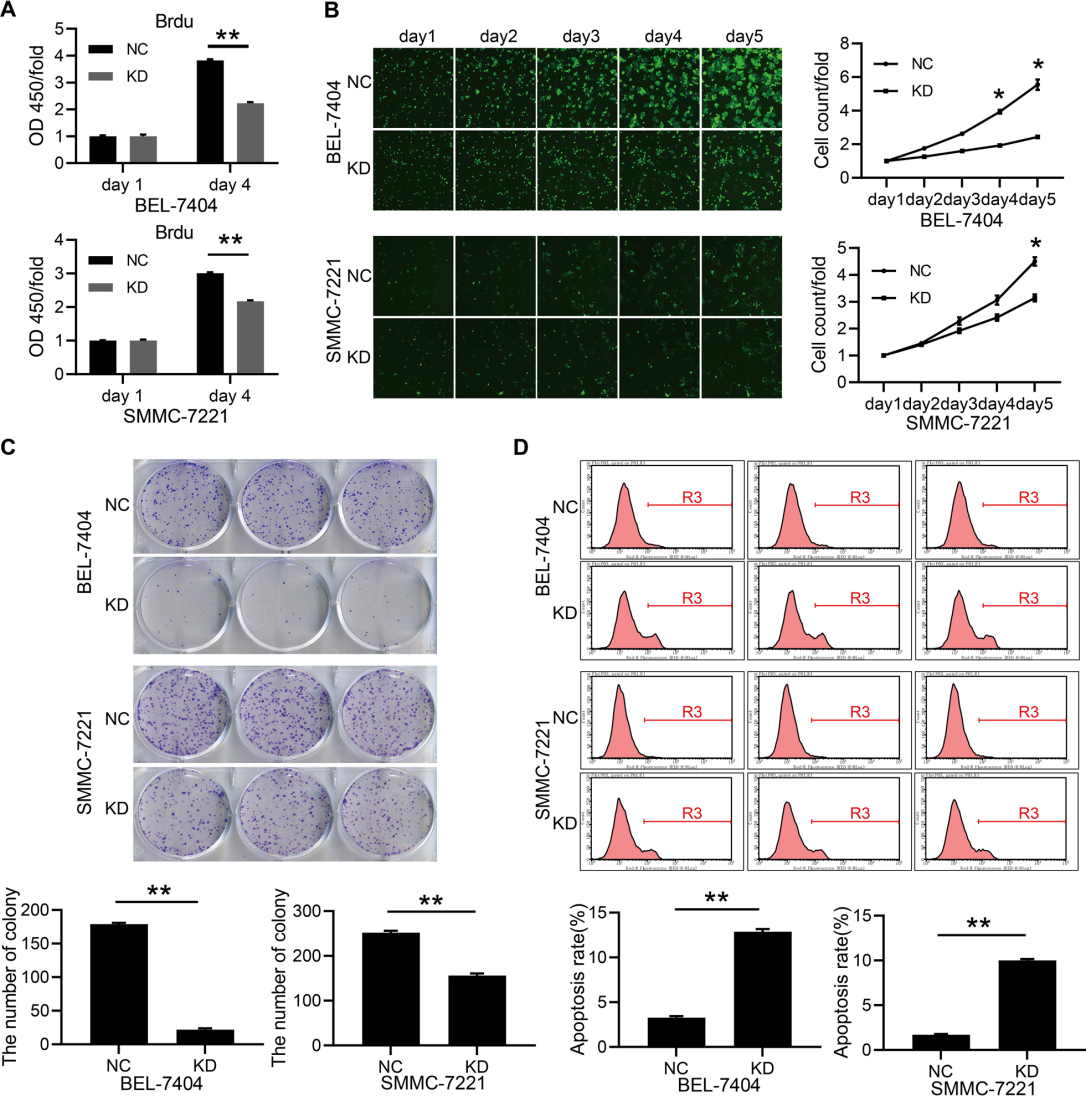
The knockdown of RSPH14 inhibited the proliferation and promoted the apoptosis of HCC cell lines. **A** Brdu assay. **B** Celigo image cytometry. **C** Colony-formation assay. **D** Flow cytometry analysis of cell apoptosis. All images with green fluorescence were scanned at ×100 magnification. All experiments were performed three times. NC: negative control; KD: RSPH14 knockdown; HCC: hepatocellular carcinoma. *P < 0.05, **P < 0.01, ***P < 0.001

After lentiviral transfection, the cell counts of BEL-7404 and SMMC-7221 cells were decreased according to the BrdU assay (P < 0.05) (Fig. 2A). In addition, the results of the Celigo image cytometry showed that the proliferation ability of transfected BEL-7404 and SMMC-7221 cells was inhibited by RSPH14 knockdown (P < 0.05) (Fig. 2B). A colony formation assay was performed to assess the role of RSPH14 knockdown on colony-forming ability. The number of colonies from the KD (RSPH14 knockdown) group was significantly decreased compared to the NC (negative control) group (P < 0.05) (Fig. 2C), indicating that RSPH14 knockdown was closely associated with the colony-forming ability of BEL-7404 and SMMC-7221 cells. These findings revealed that knockdown of RSPH14 inhibited the proliferation of HCC cells.

**Fig. 3:**
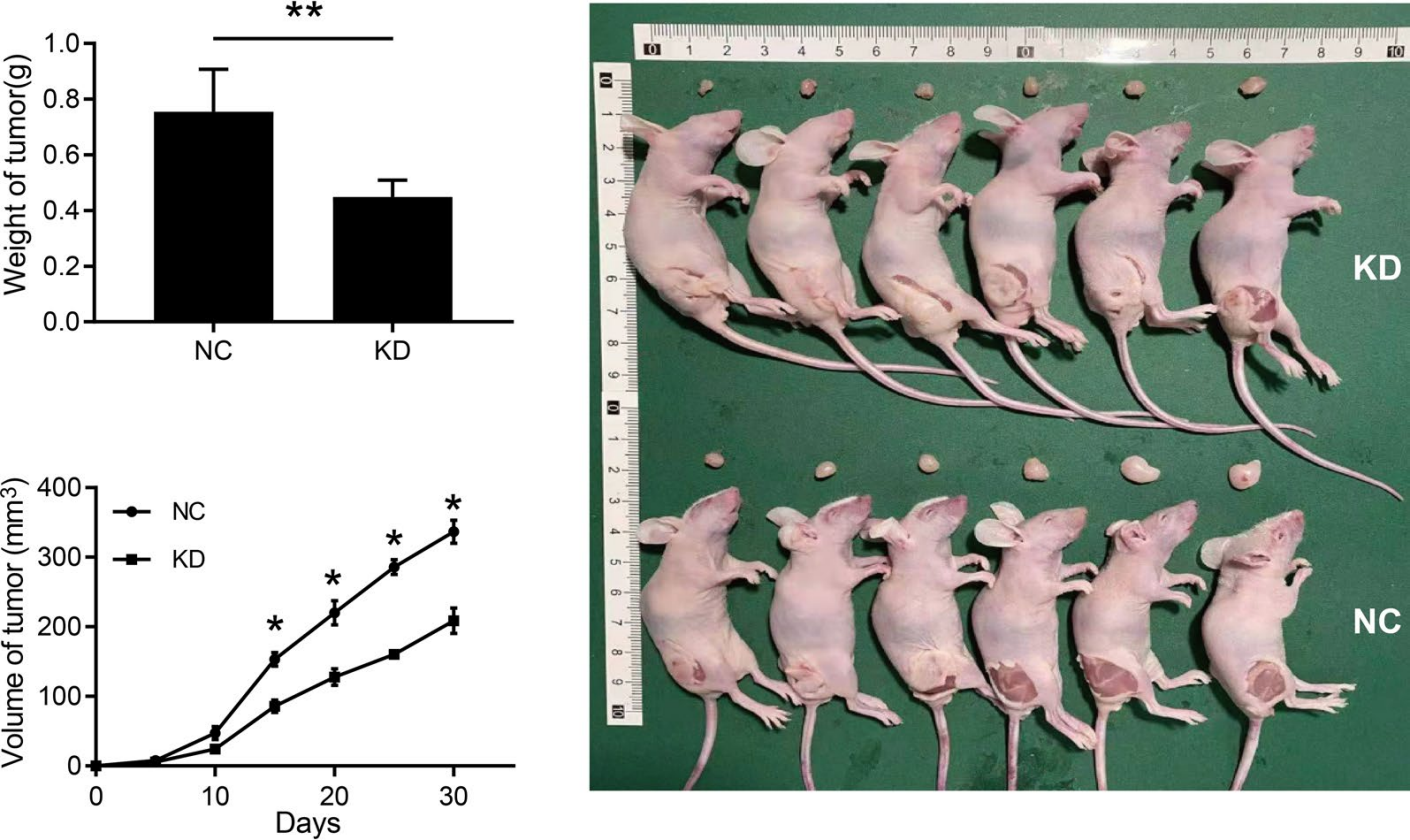
In vivo experiment confirmed that knockdown of RSPH14 inhibited tumor growth, based on the weight and volume of the tumors. NC: negative control; KD: RSPH14 knockdown. *P < 0.05, **P < 0.01, ***P < 0.001

In order to further confirm the biological function of RSPH14 in HCC, we performed an in vivo tumorigenicity assay by using a xenograft mouse model. Animals were randomly divided into two groups, and then HCC cells with or without RSPH14 knockdown (1 × 10^6^) were injected subcutaneously in their right flanks. As shown in Fig. 3, RSPH14 knockdown significantly decreased tumor growth and resulted in lower tumor weight and volume (P < 0.001). These data demonstrated that RSPH14 functions as a tumor promoter in HCC in vivo.

We also performed flow cytometry to assess apoptosis in HCC cells. After 3 days of lentivirus transfection with shRNA against RSPH14, the percentage of apoptotic cells in the KD group was significantly increased compared with the NC group (P < 0.05) (Fig. 2D), suggesting that RSPH14 depletion could induce the apoptosis of HCC cells.

### RSPH14 knockdown inhibits the migration and invasion of HCC cells

**Fig. 4:**
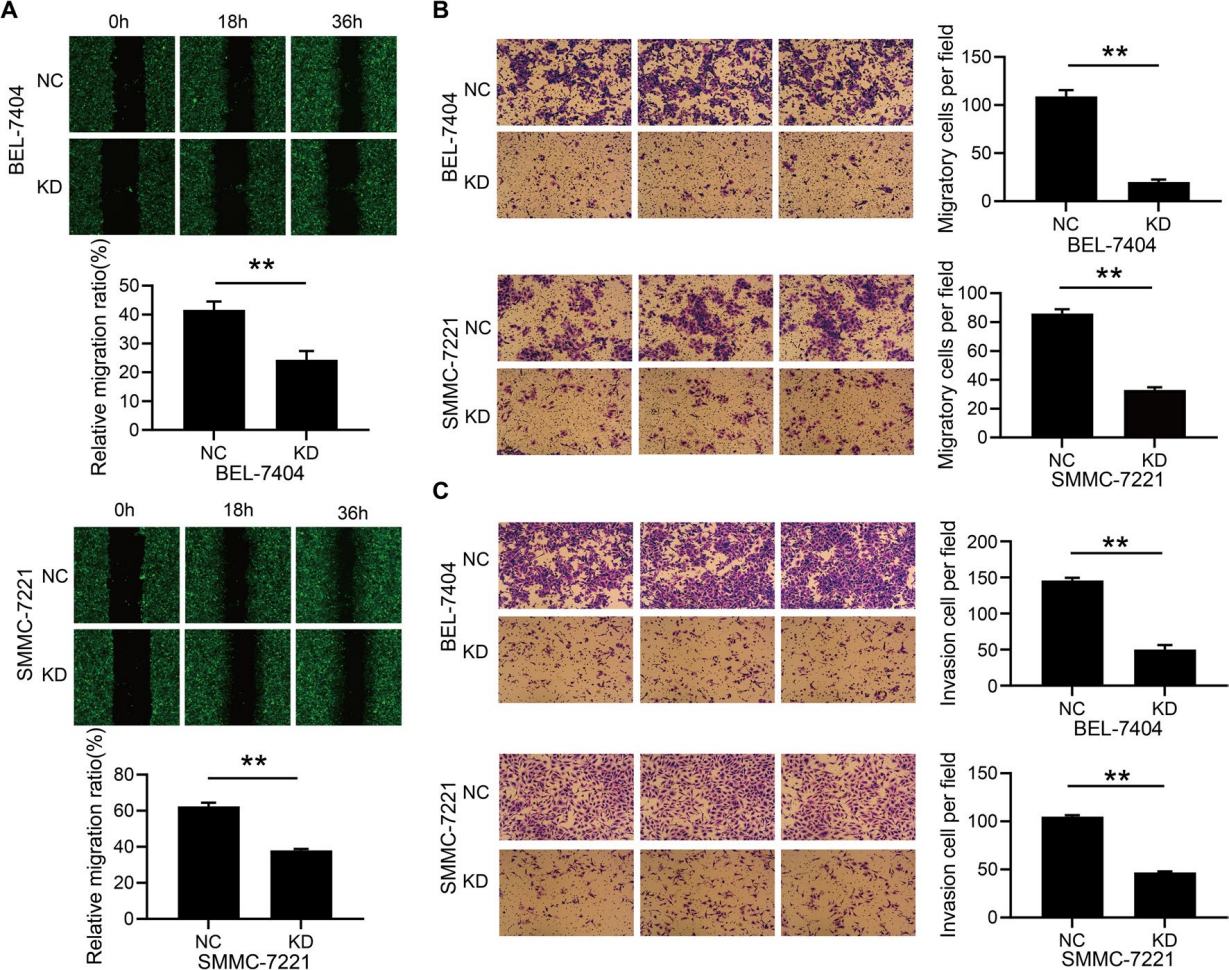
RSPH14 knockdown inhibited the migration and invasion of HCC cell lines. **A** Scratch wound-healing assay. **B** Transwell migration assay (without ECM). **C** Transwell invasion assay (with ECM). All images were scanned at ×100 magnification. All experiments were performed three times. NC: negative control; KD: RSPH14 knockdown; HCC: hepatocellular carcinoma; ECM: extracellular matrix. *P < 0.05, **P < 0.01, ***P < 0.001

Since RSPH14 depletion inhibited the proliferation and promoted the apoptosis of HCC cells, the effect of RSPH14 depletion on the migratory and invasive ability of HCC was evaluated. The wound-healing assay showed that the migration area in the KD group was decreased compared with the NC group (Fig. 4A). At 36 h after scratches, the migratory ability of BEL-7404 and SMMC-7221 cells in the KD group was significantly inhibited (P < 0.05) (Fig. 4A). Similarly, the result of the Transwell migration assay indicated that RSPH14 depletion could inhibit the migration of HCC cells (P < 0.05) (Fig. 4B). We also performed the Transwell invasion assay to investigate the role of RSPH14 knockdown on the invasion of cells. It was found that the invasion ability of BEL-7404 and SMMC-7221 cells was significantly suppressed by RSPH14 knockdown (P < 0.05) (Fig. 4C). Collectively, the migration and invasion of BEL-7404 and SMMC-7221 were inhibited by RSPH14 knockdown.

### RSPH14 knockdown suppresses the proliferation and migration of HCC cells by inhibiting RelA

**Fig. 5:**
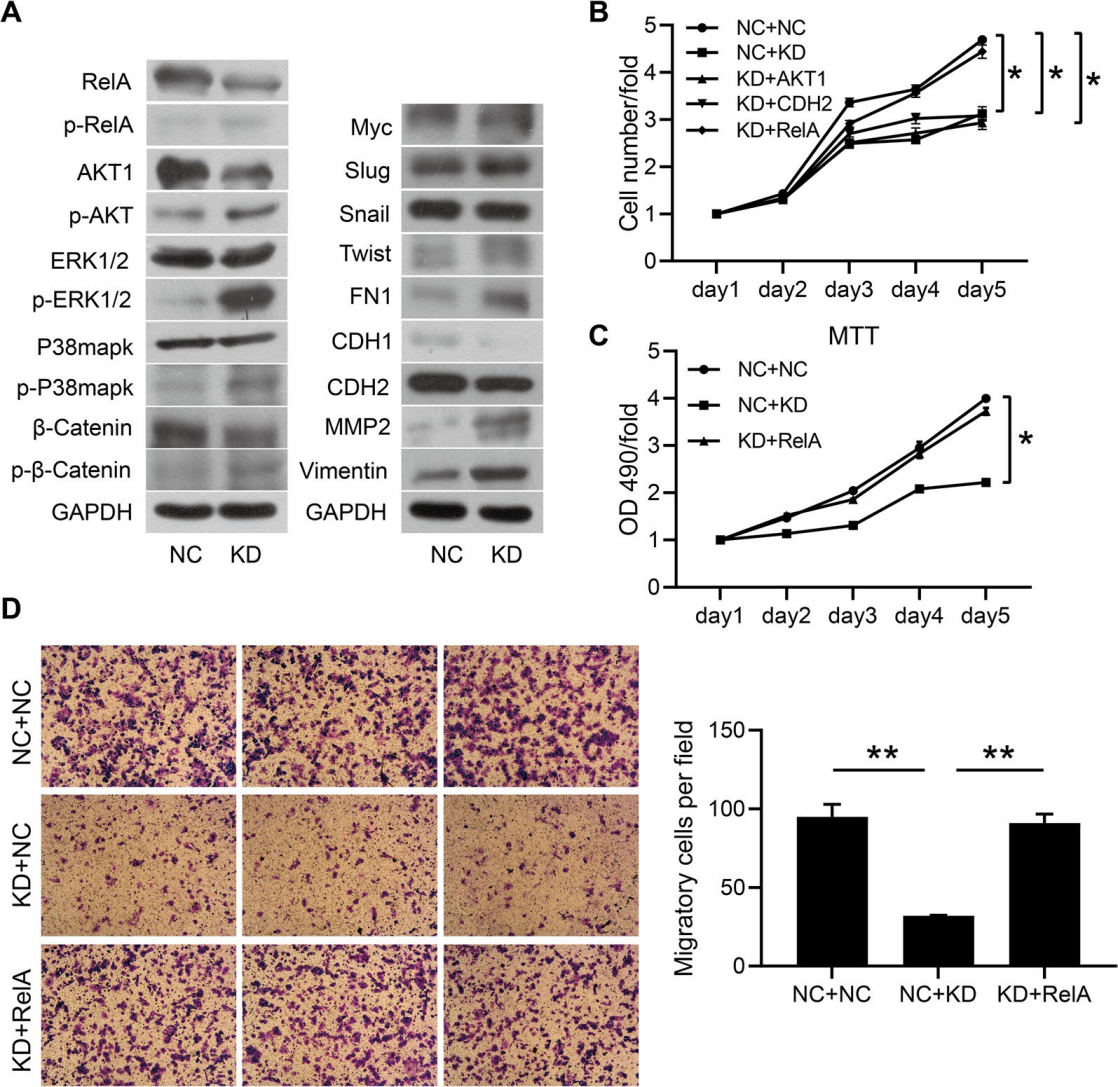
RSPH14 knockdown inhibited the proliferation and migration of HCC cell lines by suppressing RelA expression. **A** Western blot analysis. **B** HCS assay. **C** MTT assay. **D** Transwell migratory assay. The images were scanned at ×100 magnification. All experiments were performed three times. NC + NC: negative control + negative control; KD + NC: RSPH14 knockdown + negative control; KD + AKT1: RSPH14 knockdown + AKT1 overexpression; KD + CDH2: RSPH14 knockdown + CDH2 overexpression; KD + RelA: RSPH14 knockdown + RelA overexpression; HCC: hepatocellular carcinoma; HCS: high-content screening. *P < 0.05, **P < 0.01, ***P < 0.001

In order to explore the intrinsic mechanism of the effect of RSPH14 knockdown on HCC cells, we conducted western blot assays to analyze whether RSPH14 knockdown could affect the key molecules involved in HCC tumorigenesis-associated signaling pathways, apoptosis, and EMT. We found that the expression levels of 14 proteins (RelA, p-RelA, AKT1, p-AKT1, p-ERK1/2, p-P38mapk, p-β-Catenin, Myc, Twist, FN1, CDH1, CDH2, MMP2, and Vimentin) were changed after RSPH14 knockdown (Fig. 5A). Among these 14 proteins, the downregulation of the expression levels of RelA (NF-κBp65), CDH2, and AKT1 was consistent with the theoretical trend [19–21].

We further verified the roles of RelA, CDH2, and AKT1 in HCC cells. By conducting an HCS proliferation assay, we found that the inhibition of RSPH14 knockdown on BEL-7404 cells with RSPH14 knockdown and RelA overexpression was significantly reduced, compared to the BEL-7404 cells with RSPH14 knockdown, while CDH2 and AKT1 were not reduced (Fig. 5B). Moreover, the MTT assay showed that RelA overexpression could abate the inhibitory effect of BEL-7404 cell proliferation caused by RSPH14 depletion, indicating that RSPH14 knockdown could inhibit the proliferation of HCC cells by down-regulating the expression of RelA (Fig. 5C). Furthermore, the results of the Transwell migration assay confirmed that RSPH14 knockdown and RelA overexpression could significantly restrain the inhibitory effect of RSPH14 knockdown on the cell migratory ability of BEL-7404 cells, indicating that RSPH14 depletion could inhibit the cell migration by suppressing the expression of RelA (Fig. 5D). Taken together, RSPH14 depletion inhibits the proliferation and migration of HCC cells by downregulating the expression of RelA.

## Discussion

Liver cancer was the third leading cause of cancer death in the world in 2020 [1]. However, there are still many problems and challenges to be solved in the treatment of HCC. Therefore, it is necessary to explore the pathogenesis of HCC in-depth and find more effective systematic treatments. High RSPH14 expression appears to be related to poor prognosis of HCC according to data from the Human Protein Atlas [6], but the mechanisms and exact associations are unknown. Therefore, this study aimed to investigate the possible roles of RSPH14 in the proliferation, apoptosis, and invasion of HCC cells. The results suggest that knockdown of RSPH14 could modulate decrease cell proliferation, migration, and invasion and increase apoptosis of HCC cells by inhibiting RelA expression. RSPH14 could be a new treatment target for HCC. The identification of novel targets is essential to improve the prognosis of patients with HCC.

RSPH14 is recognized as a prognostic marker in renal cancer [6]. A database analysis suggested that high RSPH14 expression is associated with poor prognosis of patients with liver cancer [6], supporting the present study. Still, no previous studies reported the role of RSPH14 in HCC tumorigenesis and development. In this study, we first demonstrated that RSPH14 played an oncogenic role in human HCC. Indeed, we found the antitumor effects of RSPH14 knockdown in HCC and preliminarily explored the underlying molecular mechanisms. Down-regulation of RSPH14 could suppress the growth, migration, and invasion of HCC cell lines by inhibiting RelA. This inhibitory effect was also demonstrated in a BALB/c nude mouse xenograft tumor model.

NF-κB is a key regulator of inflammation and cell death and participates in developing a hepatocellular injury, liver fibrosis, and HCC [10, 22]. NF-κB is recognized as a potential target for the prevention and treatment of HCC. As one of the most important members of the NF-κB family, RelA (p65) combines with p50 to form the most common NF-κB heterodimer that regulates gene transcription [23]. RelA contains C-terminal transactivation domains and might be a strong activator for the transcription of genes with κB sites [24]. Therefore, RelA seems to be a crucial molecule contributing to the occurrence and progression of HCC. In this study, the knockdown of RSPH14 could significantly suppress the expression of RelA, suggesting that both RSPH14 and RelA are involved in hepatic oncogenesis. Further study demonstrated that the inhibition of cell proliferation and migration caused by RSPH14 knockdown could be reversed by RelA overexpression, demonstrating that RSPH14 is involved in regulating cell functions of HCC by modulating the expression of RelA. These results revealed the critical role of RelA in HCC development, which is supported by previous studies [10, 22, 24].

**Fig. 6:**
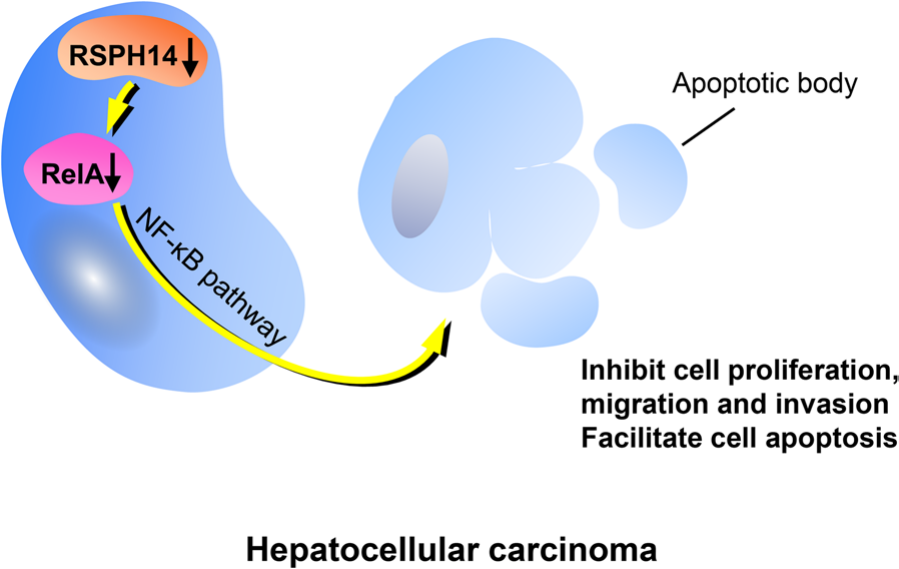
RSPH14 is involved in the growth of HCC by regulating RelA expression. HCC: hepatocellular carcinoma

The above results indicated that the down-regulation of RSPH14 or targeting the RSPH14-RelA axis might inhibit the tumor growth and invasion (Fig. 6). It could perhaps provide an alternative treatment strategy for patients with advanced HCC and improve the prognosis of patients. Future studies should aim to discover inhibitors of RSPH14 and test them in a preclinical setting.

There are still several limitations in this study. First, the mechanism of RSPH14 regulating RelA expression has not been completely elucidated since only 14 proteins were examined. Second, the role of the RSPH14-RelA axis has not been clarified in xenograft tumor models. Nevertheless, this study demonstrated the important role of RSPH14 in HCC pathogenesis.

In conclusion, RSPH14 is involved in the proliferation, apoptosis, migration, and invasion of HCC by regulating RelA expression. High RSPH14 expression is closely related to poor clinical outcomes of HCC patients. Therefore, targeting RSPH14 might provide new insights for anti-HCC treatment.

## Supplementary Information

**Additional file 1: Figure S1.** Effect of lentivirus transfection on HCC cell lines. (A) Results of lentivirus transfection in BEL-7404 and SMMC-7221 cells. The images were scanned at 100× magnification. (B) Western blot analysis. All experiments were performed three times. NC, negative control; KD, RSPH14 knockdown; HCC, hepatocellular carcinoma. *P < 0.05, **P < 0.01, ***P < 0.001.

## Data Availability

The datasets used and/or analysed during the current study are available from the corresponding author on reasonable request.

## Abbreviations

HCC: Hepatocellular carcinoma
RSPH14: Radial Spoke Head 14 Homolog
RTDR1: Rhabdoid tumor deletion region gene 1
EMT: The epithelial to mesenchymal transition
CDH1: E-cadherin
CDH2: N-cadherin
OS: Overall survival rate
PFS: Progression-free survival
