# Supplementary figures and images for: Knockdown of RSPH14 inhibits proliferation, migration, and invasion and promotes apoptosis of hepatocellular carcinoma via RelA
Source: Cancer Cell Int. 2022 Mar 19;22:129. doi: 10.1186/s12935-022-02515-z (PMC8933878; doi:10.1186/s12935-022-02515-z)

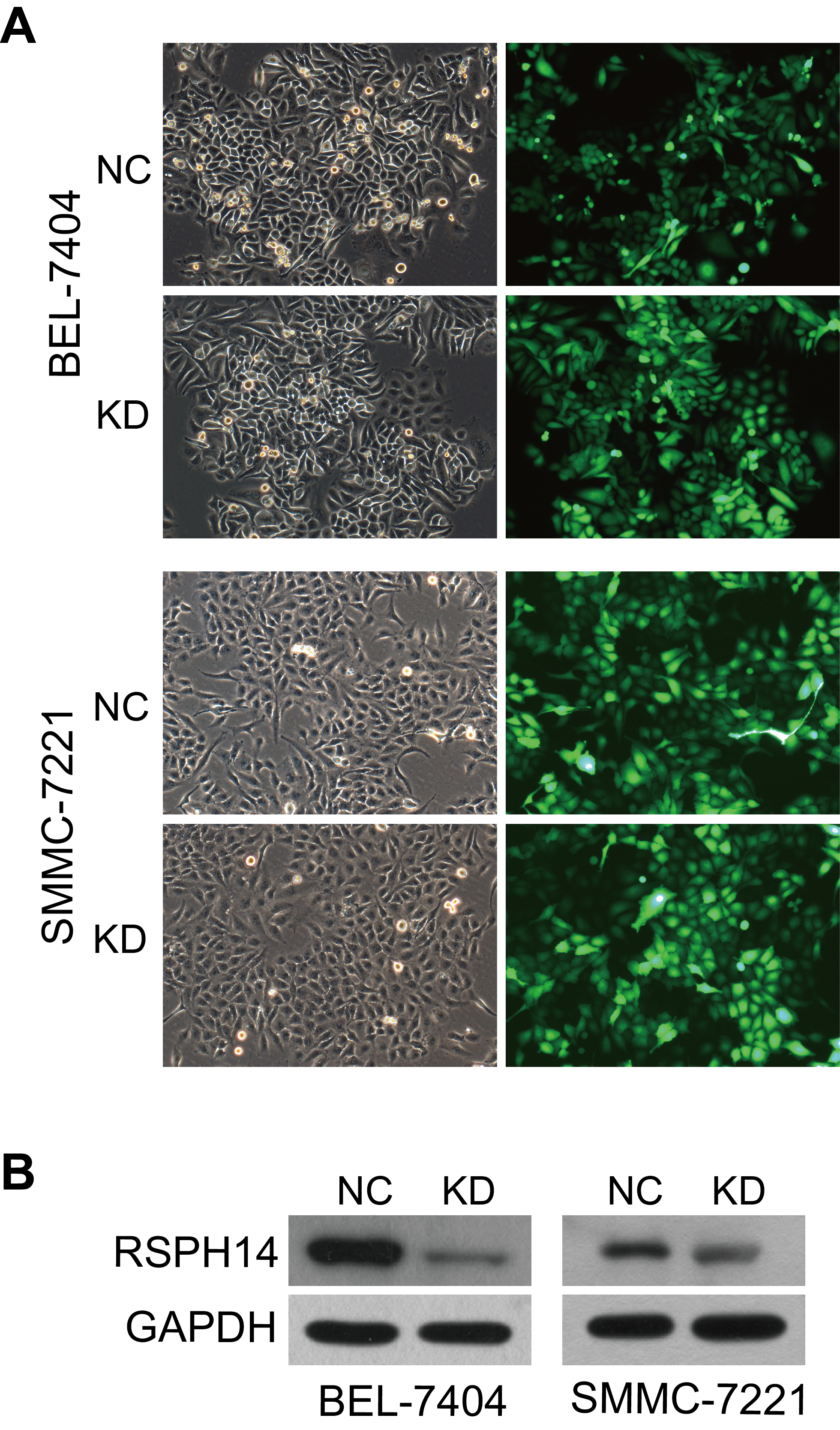

Supplement: Supplementary file 1 — Additional file 1: Figure S1. Effect of lentivirus transfection on HCC cell lines. (A) Results of lentivirus transfection in BEL-7404 and SMMC-7221 cells. The images were scanned at 100× magnification. (B) Western blot analysis. All experiments were performed three times. NC, negative control; KD, RSPH14 knockdown; HCC, hepatocellular carcinoma. *P < 0.05, **P < 0.01, ***P < 0.001. [file 12935_2022_2515_MOESM1_ESM.png]
